# Influence of Healthcare-Associated Factors on the Efficacy of Hepatitis C Therapy

**DOI:** 10.1100/2012/580216

**Published:** 2012-12-27

**Authors:** Mohamed A. Daw, Aghynya A. Dau, Mohamed M. Agnan

**Affiliations:** ^1^Department of Medical Microbiology and Immunology, Faculty of Medicine, Tripoli CC 82664, Libya; ^2^Department of Intervention Surgery, Faculty of Medicine, Tripoli CC 82664, Libya; ^3^Department of Pharmacology, Faculty of Medical Technology, Al Jabal Al Gharbi University, Naloot, Gheraian, Libya

## Abstract

Hepatitis C infection is a complex entity associated with sizable morbidity and mortality, with great social and economic consequences that put a heavy potential burden on healthcare systems allover the world. Despite the great improvement of hepatitis C virus (HCV) therapy and its high clinical efficacy, major influencing factors are still hindering and diminishing the effectiveness of hepatitis C treatment. This minimizes the quality of life of the infected patients and reduces the outcome of such therapy, particularly in certain groups of patients such as intravenous drug users and patients coinfected with human immune deficiency virus (HIV). A variety of factors were evolved either at patient individual level, healthcare providers, community surrounding levels, or healthcare setting systems. Analyzing and understanding these factors could help to improve HCV interventions and, thus, reduce the burden of such infection. The objectives of this paper were to highlight such factors and outline the holistic approaches that could be used to overcome such factors.

## 1. Introduction

Hepatitis C has been considered one of the most evolving pathogens in recent decades. Considerable advances have been made in our understanding of such virus form all aspects including epidemiology, mode of transmission, and advances in clinical management and antiviral therapy [1–3].

 Profound changes have been observed in the progress of the disease and the nature of newly infected patients [4]. The mode of transmission has been associated with a gradual reduction in the proportion of cases related to transfusion and an increase in the proportion related to intravenous drug use [4]. These changes are accounted for the emergence of other changes in genotype profiles which are characterized with the prevalence of genotype 3 which was associated with better response to treatment [5]. 

 Major advances have been made in treatment of chronic HCV with the introduction of pegylated interferon (PEG IFN) which, in combination with ribavirin, gives an overall rate of sustained virological response up to 80% for HCV genotypes 2 and 3 [5, 6]. Further to the progress in the management of coinfected patients by both HIV and HCV which have transformed the prognosis of HIV disease [7]. Major steps to be taken to make such achievement accessible to all risk groups vulnerable to such infections. The shift in trends of HCV epidemiology towards injecting drug users (IDUs) being the largest group of persons infected with the hepatitis C virus (HCV) in the world has markedly resulted in the introduction of new approaches in the management of such group [8]. This should include introduction of replacement therapy and community-based holistic approaches and concepts. 

 Complications of HCV are expected to increase tremendously over the coming decades [2, 6]. This will place a heavy burden on healthcare systems. Hence caring for hepatitis C-infected patients represents a major challenge to health-care team. That, however, requires patience, experience, and an understanding of the dynamics and circumstances that these patients have been in. Furthermore, compound and integrating programs should be implemented in caring for such patients as hepatitis C infection is never to be considered as a single entity disease, particularly among certain categories of patients as IDUs and those coinfected with HIV [7]. Therefore controlling and treating hepatitis C infection require developing and implementing a meticulous and comprehensive strategies for prevention, care, and effective treatment of HCV. Substantial variable factors such as social, economic, and other barriers have been found to influence the effective care and treatment of HCV, yet only in the last decade or so has rigorous research been conducted to better understand such factors that play major roles in population infected with HCV. Healthcare providers undertaking HC care should draw a major role in elucidating the importance of such factors. Thus, identifying and working to overcome barriers to HCV treatment have become important goals in approaching the HCV epidemic. The objectives of this paper is to address the influence of social, economic, and other factors on the efficacy of HCV therapy. 

## 2. Therapeutic Management of Hepatitis C Infection

 The sole objective of medical intervention in HCV therapy is suppression of the virus, the prevention of viral resistance, and the maintenance of the health of the patient [9, 10]. However, these goals may or may not coincide with those of the patient. Despite advancements in the management of chronic hepatitis C to achieve such goals, the target is far away to be reached as many obstacles associated with patients as well as healthcare services providers stand as major barriers [11–13]. Despite treatment of recently acquired hepatitis C can lead to sustained virological response (SVR) rates of up to 98% [9–11], many patients did not even initiate therapy and others failed to fulfill the criterions of such therapy [14, 15]. Furthermore, there continues to be a low rate of treatment uptake among certain categories of patients prone to HCV infection particularly current IDUs [16].

 The ongoing standard model of treatment for HCV infection is combination therapy with peginterferon *α*-2a or *α*-2b and ribavirin; such treatment has been associated with sustained viral response (SVR) rates of approximately 54–63% in previously untreated patients [17–19]. Response to and duration of the treatment vary according to the genotype involved. In patients with genotype 2 or 3 HCV infection, a 24-week course of peginterferon and ribavirin induces SVR in nearly 80% of patients. This however is not the case of patients infected with genotypes 1 and 4 as they do require a longer time, at least 48 weeks of treatment, and most of them (50–60%) do not attain SVR [20].

 Hence, new therapeutic agents have been tested in recent years that directly target HCV. The direct-acting antivirals (DAAs) that have been mostly studied are the protease inhibitors, telaprevir, and boceprevir, which inhibit the HCV enzymes NS3/NS4 and NS3, respectively, causing a disruption of HCV replication [21]. Further to polymerase NS5B, NS5A, entry inhibitors, or other drugs like cyclophilin inhibitors, new interferons, immune modulators, or therapeutic vaccine, other factors have been found to influence such applicable therapy. However, combination of these agents with peginterferon and ribavirin has demonstrated significant efficacy in treatment-naive patients with genotype 1 HCV infection [22]. Recent studies have investigated the best way of applying such models of therapy either by optimizing such regimens, their duration, and/or identifying factors associated with response to therapy. Indeed different factors have been found to influence and limit the effectiveness of such expensive therapy, these to be taken in to consideration in dealing with HCV intervention [23].

## 3. Factors Influencing the Efficacy of Hepatitis C Therapy 

 Increasing the proportion of HCV-infected patients particularly those associated with IDUs who receive available treatments is challenging as they were facing multiple barriers to care about. Different studies have suggested that over 75% of patients confirmed to be infected with HCV went without or even denying treatment [24, 25]. A variety of barriers was involved either at individual level and/or healthcare provider and community surrounding levels. Such studies have highlighted the importance of such barriers and their impact on the outcome of HCV therapy. These factors to be taken with great consideration when implementing therapy either at patient individual or healthcare levels. The overarching goals to identify such potentially modifiable barriers as targets for future intervention to increase the applicability and thus the effectiveness of HCV therapy. Most of these factors were elucidated in Table 1. 

## 4. Individual Patient-Associated Factors

 Pharmacological responses, histological markers, eradication of HCV, and diminishing the progress of disease have been used as major determinants for successful HCV therapy. However, amelioration of symptoms or improvement in the quality of life of infected patients is rarely mentioned as a part of such therapy [24–27]. 

 Despite all the progress that has been made in preventing HCV worldwide, antiviral therapy, however, remains the main sole in preventing serious HVC-associated liver diseases. Hence, compiling with such treatment becomes the mile stone in curing HCV and preventing any further complications associated with. Immigration status, race, and language barriers impede access to HCV-related healthcare in developed countries. This is relevant given to the multilingual and multiracial makeup of HCV-infected patients particularly in developed regions. Hence, HCV prevention, care, and treatment program must recognize community-specific epidemiology, which varies greatly by setting and level of economic development [2, 4]. Further studies were needed regarding the effect of such variables on the SVR either independently or in combination with other factors.

Medical factors such as genetic status of the patients, progress of disease, its clinical stage, side effects, and adherence of the used therapy and coinfection with other viruses have great influence on the success of HCV therapy. Recently genetic factor has been found to influence the response of treatment among HCV-infected patients [28]. Individuals of European ancestry are more likely than those of African ancestry to attain SVR with peginterferon and ribavirin, and genetic studies have revealed that approximately half of this difference is explained by a polymorphism near the *interleukin- (IL-) 28B *gene, which encodes interferon-*&*3 [29, 30]. Thus, further studies should focus on assessing the role of IL-28B polymorphisms in determining response to therapy with these new agents.

 Quality of life and sexual health have been found to be diminished among patients with chronic hepatitis C. Such functioning and satisfaction are associated with the degree of hepatic fibrosis or cirrhosis [31]. Lower sexual summary scores were highly associated as well with female gender, older age, history of cholesterol medication use, and concomitant use of antidepressant or anxiolytic medications. Such deterioration in the quality of health among patients with chronic hepatitis C can be improved, at least in part, by successful antiviral therapy. Patients who achieve an SVR may feel less stigmatized and concerned about potential transmission of HCV to their sexual partners, which has been a factor associated with lower quality of life [32, 33]. Furthermore ribavirin is highly teratogenic, hence patients seeking pregnancy and receiving therapy must diligently observe two forms of birth control during treatment and for six months after stopping [34].

The effectiveness of treatment of hepatitis C with pegylated IFN-a is affected by a poor rate of acceptance and/or adherence to currently available regimens, especially in IVDU and women. Recognized barriers are female gender, young age, psychiatric illnesses, and lack of methadone substitution therapy. In a study carried by Broers et al. [35] of the five women in whom antiviral therapy was indicated (four of them were IVDU), three refused to undergo it for fear of side effects and another abandoned it after the first injection due to intolerance. Hence, gender may significantly affect the acceptance of antiviral treatment, especially among IVDU, as observed both in acute and chronic hepatitis C.

 Patient and physician should make decisions about treatment together, after a thorough discussion of the need for adherence to the treatment regimen and the risks of adverse effects and reinfection [36]. The patient's current and previous adherence to medical regimens and his or her mental health and risk of depression should be considered, as should access to safe injection equipment and knowledge of safe injection practices; such discussions may not lead to treatment for injection-drug users [37]. This, however, will not cover all patients as some of them, with poor adherence to treatment regimens, uncontrolled depression, or unsafe injection practices may remain obstacles to therapy. Hence, further studies are needed to overcome such barriers.

## 5. Social and Environmental Factors

 Poor social circumstances, environmental barriers, and incarceration-poor housing may further compromise a person's desire and ability to seek care. Factors such as these have been shown to sustain the use of antiretroviral treatments for HCV-infected patients [38]. Therefore, one important comprehensive care program is education, as many HCV patients who decline treatment do so as a consequence of misinformation related to HCV disease and/or treatment. Furthermore, HCV incidence is greater where structural factors like poverty, stigma, or lack of services impede individuals from protecting themselves [4, 26]. Hence heuristic social models that account for the dynamic and interactive nature of structural factors that may impact HCV prevention behaviors should be designed and implemented to account for social factors associated with HCV particularly with other concomitant infections such as HIV.

HCV infection is associated with exposures thought to be common among prisoners, but it has also been suggested that incarceration itself may pose a risk for infection [39]. The prevalence of HCV infection ranges from 22% to 40% among incarcerated populations [40, 41], but few studies have examined associated risk factors, such as IDU exposure, incarceration patterns, and other high-risk behaviors. Assessment of the sources of risk for prisoners will facilitate decision making about how to screen for HCV, prevent further spread of the disease, and provide appropriate care to inmates [39]. The prevalence of HCV among incarceration groups is more fivefold than general population. Furthermore, multiple incarcerations and length of incarceration may be associated with higher risk or may be proxies for risk-taking behavior, such as unreported IDUs [40]. Hence the development of policies for systematic HCV screening among all persons entering and within the corrections system should be implemented. This could improve resource planning, education, and healthcare within correction systems and for parolees reentering the community. Furthermore, correctional facilities may actually be a more appropriate and realistic setting for treatment of this high-risk population than the general community, where healthcare is fragmented and access to it is limited.

## 6. Economic Factors Associated with Hepatitis C Infection 

 The effect of hepatitis C treatment response on medical costs has not been well studied. HCV infection, however, costs the healthcare system in developed countries a heavy burden and that is expected to be doubled in the near future [4]. The average lifetime cost (i.e., medical costs and economic losses) for an affected patient has been estimated to one $ million. Such medications may include costs for prescription, nonprescription, and complementary medications which maybe related to antiviral therapy, adverse effect and other comorbid conditions. Financial constraints, however, may lead to lack of persistence and adherence with medication use, poor health outcomes, and higher overall healthcare costs [42]. Healthcare profession hence should be vigilant for such factors and use specific strategy to help the patients to overcome such barriers [4, 28]. Further studies are needed to explore the cost effectiveness of the medication with special attention to the costly ones. 

## 7. Influence of Alcohol on Hepatitis C Infection 

 Alcohol intake has a major effect in liver-associated diseases particularly viral hepatitis. Such consumption may not only damage the liver itself but also influence the behavior of such patients. Individuals with HCV continue to seriously jeopardize their health by using and abusing alcohol. These individuals experience more rapid disease progression and more-related complications as a result of alcohol use [43]. HCV-infected people who use alcohol excessively may also engage in risky sexual behaviors while, under its influence, exposing both themselves and their partners to sexually transmitted infections [44]. Different studies have shown that the use of various substances can have an effect on antiviral medication adherence [45]. Although not all studies examining the relationship between alcohol use and medication adherence, such speculation still exists and further studies are needed to clarify such correlation.

 Alcohol has great concern in HCV patients coinfected with HIV with the consequence that end-stage liver disease accelerated as a result of alcohol use among those coinfected and accelerates the illness and death among these individuals [46]. Furthermore, HCV treatment is less effective in people with HIV coinfection, and its effectiveness is limited even more by ongoing alcohol use.

## 8. Psychiatric Factors Associated with Hepatitis C Infection 

 Psychosocial factors were considered to be the most common contraindications for antiviral therapy of patients with hepatitis C [47]. Psychiatric comorbidities are common among such patients and particular attention must be paid to mental health conditions, which are associated with both hepatitis C and substance use and may be induced or exacerbated by treatment for hepatitis C [48, 49].

 Neuropsychiatric changes are mainly reported to occur during antiviral therapy for chronic HCV infection, but there is evidence that mental health changes such as depression or cognitive disturbances may persist or even newly appear after antiviral treatment of chronic hepatitis C was finished [50]. Different studies have shown that preexisting depressive symptoms, psychiatric disorders, or drug addiction are considered as risk factors for psychiatric side effects during, or negative mental health changes after, antiviral treatment with IFN-a [51, 52]. However, long-term effects of IFN-a on mental health in patients with or without psychiatric risk factors are lacking and further studies are needed to clarify such effects.

 Few studies have examined HCV treatment outcomes within psychiatric populations. A study carried on 33 HCV-infected veterans treated with antiviral therapy for six months found that, despite similar virological response rates, 32% of patients with psychiatric comorbidities developed severe neuropsychiatric side effects that led to antiviral therapy discontinuation, compared with only 14% of patients without psychiatric comorbidities [6, 53]. Therefore, patients should be screened for depression and other mental health problems before beginning treatment with IFN, treated if necessary, and monitored for these problems during treatment for HCV. Some investigators have recommended prophylactic antidepressant therapy before beginning treatment for HCV in patients who were thought to have a high risk of depression [23, 54].

In a recent study Huckans et al. [55], compared rates of psychiatric symptoms in patients with schizophrenia who were receiving antiviral therapy for HCV to rates of psychiatric symptoms among patients with schizophrenia and HCV who were not receiving antiviral therapy suggesting that patients with schizophrenia experience similar rates of psychiatric symptoms on and off antiviral therapy. Such data indicated that schizophrenia is not a contraindication to antiviral therapy for HCV. Hence further detailed studies are needed on patient monitoring during treatment which focus on drug efficacy and tolerability (with special attention to psychiatric disorders) and on quality of life particularly among risk groups who prone to needle phobia.

## 9. Factors Associated with HIV Patients Coinfected with HCV

 Due to common risk factors for exposure, HIV and HCV are often found concurrently up to 30% of HIV-positive patients who are coinfected with hepatitis C including 70%–80% of IDUs, and HCV-related liver disease is a leading cause of death in this population [56]. Yet studies consistently show that less than one-third of HIV coinfected patients in the United States are deemed eligible for HCV treatment, and less than 10% actually receive treatment [57]. Upon detection of HCV infection, for treatment to be provided, providers must first consider a patient as appropriate treatment candidate, and multiple medical and psychosocial factors can contribute to a provider's reluctance to recommend or offer treatment to a patient [58, 59]. HIV negatively impacts HCV-induced liver disease, resulting in accelerated progression to cirrhosis, liver failure, and liver-specific death. In spite of these potential benefits, the majority of HIV-HCV coinfected patients do not initiate HCV antiviral therapy as a consequence of multiple concurrent barriers to care [60]. Furthermore, HIV infection itself has been identified as a potential barrier to healthcare [59]. However, SVR is diminished in HIV-HCV coinfection independent of language barrier, race, immigration status, or socioeconomic status. Healthcare infrastructure that can provide great support to HCV coinfection to improve the quality of life should be formulated. This can be achieved in spite of the presence of such well-established barriers to healthcare provision and outcomes. 

## 10. Factors Associated with Injecting Drug Users 

 Injection drug users constitute the largest group of persons infected with HCV particularly among the developed countries, and even most the new infections occur in this group [61, 62]. The prevalence of HCV among IDUs reached up to 90% and uninfected IDUs generally become infected at rates of 10–20%/year [63, 64]. Intravenous drug users are a major risk group for infection with HCV [3, 4] and globally approximately 90% of newly acquired HCV infections are caused due to sharing of injection equipment among IVDUs [65]. Treatment of IVDU with chronic hepatitis C has been generally discouraged, for presumed nonadherence, increased risk and/or severity of side effects (especially psychiatric), and risk of reinfection [66]. On the other hand, IVDUs often share many factors of good response to therapy, such as young age, short duration of disease, and HCV genotype 3 infection. Moreover, different clinical studies suggest that IVDU can be treated successfully also when active use of drugs has been withdrawn for only a short period of time [16, 67, 68]. Thus, more effective guidelines contain less restrictive recommendations.

There was considerable variation in SVR rates among IDUs in different trials, ranging from 15.8% to 94.1% for chronic hepatitis C and from 50.0% to 100.0% for acute hepatitis C. This variation may be attributable to variations in the study designs (e.g., recruitment criteria) and variations in the treatment of participants (e.g., treatment regimen or psychosocial support). Nonetheless, when combined, the results from these trials provide the broadest and most rigorous account to date of hepatitis C treatment outcomes in IDUs [69, 70]. Nonetheless, programs are successfully integrating hepatitis C care for IDUs into health-care settings, including primary care, methadone treatment and other substance-abuse treatment programs, infectious disease clinics, and clinics in correctional facilities [71, 72]. Despite the difficulties that may oppose the treatment of such vulnerable group, it is important to know that the support of an aggressive management of acute hepatitis C in active IVDU is not only to prevent these patients from progressing to chronically evolving liver disease, but also to curb the HCV spread within the IVDU community.

## 11. Strategies and Future Aspects 

 Hepatitis C infection is expected to increase dramatically in the coming years particularly among young and dynamic sectors of the society. This is clearly evident by the modification of HC infection epidemiology as most of the infected individuals expose themselves voluntarily by sharing needles of injecting drugs. Hence the future aspects for HCV infection should be dealt with differently away from medical problems alone. Holistic focus should be on providing ongoing support to engage and retain HCV patients with complex social and medical healthcare needs. This may include pretreatment intervention and multidisciplinary model of treatment as shown in Table 2.

 Specific support measures should be carefully evaluated and implemented as the following: they should include an individualized management and an improved education of infectious disease specialists or gastroenterologists in addiction medicine (and of addiction specialists in infectious diseases), possibly via the integration of HCV treatment within substance abuse treatment. Thus, future management of hepatitis C patients should preferably be carried out in an integrated way at highly specialized, multidisciplinary units [73]. 

 It is obvious that these factors diminishe the current rate of HCV treatment, while most of them are amenable to change. Hence efforts to overcome such barriers require patient and provider education, interventions to improve clinic attendance, and collaboration between primary care physician and both mental health and substance abuse professionals. Enhancement of cooperation and reducing the distance between all those directly or indirectly involved in HCV management by introducing HCV treatment into the primary care clinic represent another system-level strategy. A comprehensive HCV disease management model addressing each of these barriers, analogous to those that have improved disease outcomes for other prevalent conditions such as long-lasting diseases (as diabetes), may offer the most effective solution to low HCV treatment rates and may thus raise the overall efficacy of treatment [74, 75].

## Figures and Tables

**Table 1:** Factors influencing the efficacy of treatment of hepatitis C virus.

Factors associated with individual patients	
(i) Immigration status, race, language	
(ii) Medical condition and comorbidities	
(iii) Alcohols	
(iv) Patient preference	
(v) Appointments and followup	
Health care or provider-associated factors	
(i) Health services	
(ii) Reluctance and hesitation	
(iii) Treatment perceptions and contraindications	
System factors	
(i) Social factors	
(ii) Insurance coverage	
(iii) Quality of life	
(iv) Psychological wellbeing	
(v) Communications with medical specialists	
Miscellaneous (Combined) factors	
(i) Environmental factors, toxic	
(ii) Quality of life	
(iii) Social factors	

**Table 2:** Approaches to overcome factors influencing hepatitis C treatment.

Universal approaches	
(i) Pretreatment intervention scheme may be applied	
(ii) Multidisciplinary management caring team (nurse, infectious disease physician, social care worker, psychiatrist)	
(iii) High professional level of management regarding diagnosis, treatment regimens, and followup	
(iv) Educational scheme for patients	
(v) Cooperative and family-like relationship with patients regarding mutual respect, acknowledgement, avoiding frustration, palming, and expectation	
Specific approaches	
(i) Patients coinfected with HIV	
(ii) Patients with IDUs	
(iii) Women seeking pregnancy undergoing treatment	
(iv) Alcoholic patients	
(v) Patients with underlying depilated diseases	
(vi) Introduction of case-based management	

## References

[1] Pol S, Vallet-Pichard A, Corouge M, Mallet VO (2012). Hepatitis C: epidemiology, diagnosis, natural history and therapy. *Contributions to Nephrology*.

[2] Daw MA, Dau AA (2012). Hepatitis C virus in Arab world: a state of concern. *The Scientific World Journal*.

[3] Maghlaoui A (2012). Challenges and issues in managing hepatitis C. *Nursing Times*.

[4] Averhoff FM, Glass N, Holtzman D (2012). Global burden of hepatitis C: considerations for healthcare providers in the United States. *Clinical Infectious Diseases*.

[5] Barreiro P, Vispo E, Labarga P, Soriano V (2012). Management and treatment of chronic hepatitis C in HIV patients. *Seminars in Liver Disease*.

[6] Louie KS, St Laurent S, Forssen UM, Mundy LM, Pimenta JM (2012). The high comorbidity burden of the hepatitis C virus infected population in the United States. *BMC Infectious Diseases*.

[7] Taylor LE, Swan T, Mayer KH (2012). HIV coinfection with hepatitis C virus: evolving epidemiology and treatment paradigms. *Clinical Infectious Diseases*.

[8] Islam MM, Topp L, Conigrave KM (2012). Linkage into specialist hepatitis C treatment services of injecting drug users attending a needle syringe program-based primary healthcare centre. *Journal of Substance Abuse Treatment*.

[9] Talwani R, Gilliam BL, Rizza SA, Nehra V, Temesgen Z (2012). Current status of treatment for chronic hepatitis C virus infection. *Drugs of Today*.

[10] Michielsen P, Ho E, Francque S (2012). Does antiviral therapy reduce the risk of hepatocellular carcinoma in patients with chronic hepatitis C?. *Minerva Gastroenterologica e Dietologica*.

[11] McHutchison JG, Everson GT, Gordon SC (2009). Telaprevir with peginterferon and ribavirin for chronic HCV genotype 1 infection. *New England Journal of Medicine*.

[12] McHutchison JG, Manns MP, Muir AJ (2010). Telaprevir for previously treated chronic HCV infection. *New England Journal of Medicine*.

[13] Kwo PY, Lawitz EJ, McCone J (2010). Efficacy of boceprevir, an NS3 protease inhibitor, in combination with peginterferon alfa-2b and ribavirin in treatment-naive patients with genotype 1 hepatitis C infection (SPRINT-1): an open-label, randomised, multicentre phase 2 trial. *The Lancet*.

[14] Poordad F, Khungar V (2011). Emerging therapeutic options in hepatitis C virus infection. *The American Journal of Managed Care*.

[15] Gidding HF, Law MG, Amin J (2012). Hepatitis C treatment outcomes in Australian clinics. *The Medical Journal of Australia*.

[16] Dore GJ, Hellard M, Matthews GV (2010). Effective treatment of injecting drug users with recently acquired Hepatitis C virus infection. *Gastroenterology*.

[17] Yee HS, Chang MF, Pocha C (2012). Update on the management and treatment of hepatitis C virus infection: recommendations from the Department of Veterans Affairs Hepatitis C Resource Center Program and the National Hepatitis C Program Office. *American Journal of Gastroenterology*.

[18] (2002). National Institutes of Health Consensus Development Conference statement: Management of Hepatitis C. *Hepatology*.

[19] Dhumeaux D, Marcellin P, Lerebours E (2003). Treatment of hepatitis C. The 2002 French consensus. *Gut*.

[20] Mecenate F, Pellicelli AM, Barbaro G (2010). Short versus standard treatment with pegylated interferon alfa-2A plus ribavirin in patients with hepatitis C virus genotype 2 or 3: the cleo trial. *BMC Gastroenterology*.

[21] Chayama K, Takahashi S, Toyota J (2012). Dual therapy with the nonstructural protein 5A inhibitor, daclatasvir, and the nonstructural protein 3 protease inhibitor, asunaprevir, in hepatitis C virus genotype 1b-infected null responders. *Hepatology*.

[22] Negro F, Alberti A (2011). The global health burden of hepatitis C virus infection. *Liver International*.

[23] Leroy V, Serfaty L, Bourlière M (2012). Protease inhibitor-based triple therapy in chronic hepatitis C: Guidelines by the French Association for the Study of the Liver. *Liver International*.

[24] Falck-Ytter Y, Kale H, Mullen KD, Sarbah SA, Sorescu L, McCullough AJ (2002). Surprisingly small effect of antiviral treatment in patients with hepatitis C. *Annals of Internal Medicine*.

[25] Cawthorne CH, Rudat KR, Burton MS (2002). Limited success of HCV antiviral therapy in United States veterans. *American Journal of Gastroenterology*.

[26] Bonkovsky HL, Snow KK, Malet PF (2007). Health-related quality of life in patients with chronic hepatitis C and advanced fibrosis. *Journal of Hepatology*.

[27] Snow KK, Bonkovsky HL, Fontana RJ (2010). Changes in quality of life and sexual health are associated with low-dose peginterferon therapy and disease progression in patients with chronic hepatitis C. *Alimentary Pharmacology and Therapeutics*.

[28] Brown RS (2011). Recent advances in hepatitis C: highlights from the 2010 AASLD meeting. *Gastroenterology and Hepatology*.

[29] Ge D, Fellay J, Thompson AJ (2009). Genetic variation in IL28B predicts hepatitis C treatment-induced viral clearance. *Nature*.

[30] Srivastava S, Bertagnolli M, Lewis JH (2005). Sustained virological response rate to pegylated interferon plus ribavirin for chronic hepatitis C in African Americans: results in treatment-naïve patients in a university liver clinic. *Journal of the National Medical Association*.

[31] Teuber G, Schäfer A, Rimpel J (2008). Deterioration of health-related quality of life and fatigue in patients with chronic hepatitis C: association with demographic factors, inflammatory activity, and degree of fibrosis. *Journal of Hepatology*.

[32] Danoff A, Khan O, Wan DW (2006). Sexual dysfunction is highly prevalent among men with chronic hepatitis C virus infection and negatively impacts health-related quality of life. *American Journal of Gastroenterology*.

[33] Zickmund S, Ho EY, Masuda M, Ippolito L, LaBrecque DR (2003). ‘They treated me like a leper’ Stigmatization and the quality of life of patients with hepatitis C. *Journal of General Internal Medicine*.

[34] Hartwell D, Jones J, Baxter L, Shepherd J (2012). Shortened peginterferon and ribavirin treatment for chronic hepatitis C. *International Journal of Technology Assessment in Health Care*.

[35] Broers B, Helbling B, François A (2005). Barriers to interferon-*α* therapy are higher in intravenous drug users than in other patients with acute hepatitis C. *Journal of Hepatology*.

[36] Cooper CL, Giordano C, Mackie D, Mills EJ (2010). Equitable access to HCV care in HIV-HCV co-infection can be achieved despite barriers to health care provision. *Journal of Therapeutics and Clinical Risk Management*.

[37] Lo Re V, Teal V, Localio AR, Amorosa VK, Kaplan DE, Gross R Adherence to hepatitis C virus therapy in HIV/Hepatitis C-coinfected patients.

[38] Awofeso N (2010). Prisons as social determinants of hepatitis C virus and tuberculosis infections. *Public Health Reports*.

[39] Fox RK, Currie SL, Evans J (2005). Hepatitis C virus infection among prisoners in the California State correctional system. *Clinical Infectious Diseases*.

[40] Allwright S, Bradley F, Long J, Barry J, Thornton L, Parry JV (2000). Prevalence of antibodies to hepatitis B, hepatitis C, and HIV and risk factors in Irish prisoners: results of a national cross sectional survey. *British Medical Journal*.

[41] Ruiz JD, Molitor F, Sun RK (1999). Prevalence and correlates of hepatitis c virus infection among inmates entering the California correctional system. *Western Journal of Medicine*.

[42] Sanyal C, Ingram EL, Sketris IS, Peltekian KM, Kirkland S (2011). Coping strategies used by patients infected with hepatitis C virus who are facing medication costs. *Canadian Journal of Hospital Pharmacy*.

[43] Piasecki BA, Lewis JD, Reddy KR (2004). Influence of alcohol use, race, and viral coinfections on spontaneous HCV clearance in a US veteran population. *Hepatology*.

[44] Serra MA, Escudero A, Rodríguez F, Del Olmo JA, Rodrigo JM (2003). Effect of hepatitis C virus infection and abstinence from alcohol on survival in patients with alcoholic cirrhosis. *Journal of Clinical Gastroenterology*.

[45] Strauss SM, Tiburcio NJ, Munoz-Plaza C (2009). HIV care providers’ implementation of routine alcohol reduction support for their patients. *AIDS Patient Care and STDs*.

[46] Easterbrook P, Sands A, Harmanci H (2012). Challenges and priorities in the management of HIV/HBV and HIV/HCV coinfection in resource-limited settings. *Seminars in Liver Disease*.

[47] Rowan PJ, Tabasi S, Abdul-Latif M, Kunik ME, El-Serag HB (2004). Psychosocial factors are the most common contraindications for antiviral therapy at initial evaluation in veterans with chronic hepatitis C. *Journal of Clinical Gastroenterology*.

[48] Rifai MA, Gleason OC, Sabouni D (2010). Psychiatric care of the patient with hepatitis c: a review of the literature. *Primary Care Companion to the Journal of Clinical Psychiatry*.

[49] Schaefer M, Capuron L, Friebe A (2012). Hepatitis C infection, antiviral treatment and mental health: a European expert consensus statement. *Journal of Hepatology*.

[50] Sockalingam S, Abbey SE (2009). Managing depression during hepatitis C treatment. *Canadian Journal of Psychiatry*.

[51] Dieperink E, Willenbring M, Ho SB (2000). Neuropsychiatric symptoms associated with hepatitis C and interferon alpha: a review. *American Journal of Psychiatry*.

[52] Dieperink E, Ho SB, Thuras P, Willenbring ML (2003). A prospective study of neuropsychiatric symptoms associated with interferon-*α*-2b and ribavirin therapy for patients with chronic hepatitis C. *Psychosomatics*.

[53] Ho SB, Nguyen H, Tetrick LL, Opitz GA, Basara ML, Dieperink E (2001). Influence of psychiatric diagnoses on interferon-*α* treatment for chronic hepatitis C in a veteran population. *American Journal of Gastroenterology*.

[54] Musselman DL, Lawson DH, Gumnick JF (2001). Paroxetine for the prevention of depression induced by high-dose interferon alfa. *New England Journal of Medicine*.

[55] Huckans M, Mitchell A, Pavawalla S (2010). The influence of antiviral therapy on psychiatric symptoms among patients with hepatitis C and schizophrenia. *Antiviral Therapy*.

[56] Fleming CA, Craven DE, Thornton D, Tumilty S, Nunes D (2003). Hepatitis C virus and human immunodeficiency virus coinfection in an urban population: low eligibility for interferon treatment. *Clinical Infectious Diseases*.

[57] Restrepo A, Johnson TC, Widjaja D (2005). The rate of treatment of chronic hepatitis C in patients co-infected with HIV in an urban medical centre. *Journal of Viral Hepatitis*.

[58] Thompson VV, Ragland KE, Hall CS, Morgan M, Bangsberg DR (2005). Provider assessment of eligibility for hepatitis C treatment in HIV-infected homeless and marginally housed persons. *AIDS*.

[59] Hadigan C, Kottilil S (2011). Hepatitis C virus infection and coinfection with human immunodeficiency virus: challenges and advancements in management. *Journal of the American Medical Association*.

[60] Norton BL, Park L, McGrath LJ, Proeschold Bell RJ, Muir AJ, Naggie S (2012). Health care utilization in HIV-infected patients: assessing the burden of hepatitis C virus coinfection. *AIDS Patient Care and STDs*.

[61] World Health Organization Viral cancers. http://www.who.int/vaccine_research/diseases/viral_cancers/en/index2.html.

[62] Kleinman S, Busch MP, Murphy EL, Glynn SA (2012). National Heart Lung Blood Institute Retrovirus Epidemiology Donor Study; Retrovirus Epidemiology Donor Study-II.The National Heart, Lung, and Blood Institute retrovirus epidemiology donor studies (Retrovirus Epidemiology Donor Study and Retrovirus Epidemiology Donor Study-II): twenty years of research to advance blood product safety and availability. *Transfusion Medicine Reviews*.

[63] Wasley A, Grytdal S, Gallagher K (2008). Surveillance for acute viral hepatitis—United States, 2006. *Morbidity and Mortality Weekly Report*.

[64] Hepatitis C Virus Projections Working Group Estimates and projections of the hepatitis C virus epidemic in Australia 2006. http://www.nchec.org/.

[65] Hellard M, Sacks-Davis R, Gold J (2009). Hepatitis c treatment for injection drug users: a review of the available evidence. *Clinical Infectious Diseases*.

[66] Mehta SH, Genberg BL, Astemborski J (2008). Limited uptake of hepatitis C treatment among injection drug users. *Journal of Community Health*.

[67] Kurelac I, Papic N, Sakoman S (2011). Intravenous drug users can achieve a high sustained virological response rate: experience from Croatian Reference Center for Viral Hepatitis. *Hepatitis Monthly*.

[68] Jafferbhoy H, Miller MH, Dunbar JK, Tait J, McLeod S, Dillon JF (2012). Intravenous drug use: not a barrier to achieving a sustained virological response in HCV infection. *Journal of Viral Hepatitis*.

[69] Gazdíková K, Gazdík F, Kajaba I, Hučková D, Daniš D, Okruhlica L (2012). Virological sustained response to former young intravenous drug abusers with chronic hepatitis C treated by pegylated interferon-*α* plus ribavirin. *Vnitrni Lekarstvi*.

[70] Grebely J, Prins M, Hellard M (2012). Hepatitis C virus clearance, reinfection, and persistence, with insights from studies of injecting drug users: towards a vaccine. *The Lancet Infectious Diseases*.

[71] Vickerman P, Martin N, Turner K, Hickman M (2012). Can needle and syringe programmes and opiate substitution therapy achieve substantial reductions in hepatitis C virus prevalence? Model projections for different epidemic settings. *Addiction*.

[72] Grebely J, Matthews GV, Hellard M (2011). Adherence to treatment for recently acquired hepatitis C virus (HCV) infection among injecting drug users. *Journal of Hepatology*.

[73] McCaughan GW (2012). Is the end of chronic hepatitis C virus infection in sight?. *The Medical Journal of Australia*.

[74] Grant RW, Hamrick HE, Sullivan CM (2003). Impact of population management with direct physician feedback on care of patients with type 2 diabetes. *Diabetes Care*.

[75] Hellard ME, Jenkinson R, Higgs P (2012). Modelling antiviral treatment to prevent hepatitis C infection among people who inject drugs in Victoria, Australia. *The Medical Journal of Australia*.

